# Dynamic 3D Cell Rearrangements Guided by a Fibronectin Matrix Underlie Somitogenesis

**DOI:** 10.1371/journal.pone.0007429

**Published:** 2009-10-15

**Authors:** Gabriel G. Martins, Pedro Rifes, Rita Amândio, Gabriela Rodrigues, Isabel Palmeirim, Sólveig Thorsteinsdóttir

**Affiliations:** 1 Centro de Biologia Ambiental, Departamento de Biologia Animal Faculdade de Ciências, Universidade de Lisboa, Lisboa, Portugal; 2 Instituto Gulbenkian de Ciência, Oeiras, Portugal; 3 Life and Health Sciences Research Institute (ICVS), School of Health Sciences, University of Minho, Braga, Portugal; Katholieke Universiteit Leuven, Belgium

## Abstract

Somites are transient segments formed in a rostro-caudal progression during vertebrate development. In chick embryos, segmentation of a new pair of somites occurs every 90 minutes and involves a mesenchyme-to-epithelium transition of cells from the presomitic mesoderm. Little is known about the cellular rearrangements involved, and, although it is known that the fibronectin extracellular matrix is required, its actual role remains elusive. Using 3D and 4D imaging of somite formation we discovered that somitogenesis consists of a complex choreography of individual cell movements. Epithelialization starts medially with the formation of a transient epithelium of cuboidal cells, followed by cell elongation and reorganization into a pseudostratified epithelium of spindle-shaped epitheloid cells. Mesenchymal cells are then recruited to this medial epithelium through accretion, a phenomenon that spreads to all sides, except the lateral side of the forming somite, which epithelializes by cell elongation and intercalation. Surprisingly, an important contribution to the somite epithelium also comes from the continuous egression of mesenchymal cells from the core into the epithelium via its apical side. Inhibition of fibronectin matrix assembly first slows down the rate, and then halts somite formation, without affecting pseudopodial activity or cell body movements. Rather, cell elongation, centripetal alignment, N-cadherin polarization and egression are impaired, showing that the fibronectin matrix plays a role in polarizing and guiding the exploratory behavior of somitic cells. To our knowledge, this is the first 4D *in vivo* recording of a full mesenchyme-to-epithelium transition. This approach brought new insights into this event and highlighted the importance of the extracellular matrix as a guiding cue during morphogenesis.

## Introduction

Imaging morphogenesis in live embryos and tissues has revealed that cells are much more dynamic than previously thought, changing their shape and behavior in ways that our interpretation of successive static images of developmental stages could not have predicted [1]–[5]. Analysis of cell behavior *in vivo* has caused a revival of the concept that morphogenesis is generated through the modulation of mechanical properties of cells, affecting their shape and relationship with the surroundings [6]–[9]. The extracellular matrix (ECM) surrounding cells *in vivo* is a key regulator of their shape, differentiation state and motile behavior [10]–[12]. Cell engagement of the ECM through integrins (or other receptors), in turn affects the mechanical state of the cytoskeleton and often also translates the mechanical forces of the ECM into chemical signals intracellularly [7], [13]–[15]. Furthermore, cell-ECM engagement is known to modulate cell-cell adhesion, another important player in the regulation of morphogenesis [16]. Thus, to fully understand morphogenesis *in vivo* we must know how cells behave during morphogenetic events and how they interact with, and are influenced by the surrounding ECM.

Metamerization of the vertebrate axial musculoskeletal, nervous and circulatory systems is established during development as a result of the transformation of a mass of mesenchymal cells, which make up the presomitic mesoderm (PSM), into a series of epithelial somites located on both sides of the neural tube [17], [18]. Somites are formed in a periodic fashion (every 90 minutes in the chick) emerging from the rostral end of the PSM as spheres of epithelial cells organized centripetally around a mesenchymal somitocoel [19]–[21]. Much is known about the molecular mechanisms which define the periodic positioning of somitic boundaries as well as the rostro-caudal polarity of somites [22]–[24]. However, almost nothing is known about how cell shape and behavior are altered as the mesenchymal PSM transforms into epithelial somites.

A fibronectin (FN) matrix surrounds the PSM and recently formed somites [25], [26] and inactivation of the FN gene (*Fn1*) in the mouse prevents somitogenesis [27], [28]. More recently, the importance of FN in somitogenesis has also been established in zebrafish [29], [30], *Xenopus laevis* [31], [32] and chick [33]. We have demonstrated that in chick, the FN matrix surrounding the PSM is generated through collaboration between ectoderm and PSM: the ectoderm produces FN which is then assembled by integrin α5β1 expressed by PSM cells. Furthermore, when FN matrix assembly is blocked, somitogenesis fails [33]. These results explain the requirement of ectoderm for morphological somite formation [34], [35] and demonstrate that FN needs to be assembled into a matrix in order to support somitogenesis. However, in spite of these recent advances, we still do not know how the FN matrix affects the transformation of mesenchymal PSM cells into epithelial somitic cells.

To better understand somite morphogenesis *in vivo*, and how a mesenchyme-to-epithelium transition occurs in the embryo, we have improved procedures for 3D imaging both live and fixed chick embryos. We first obtained detailed images of fixed embryos revealing the cellular organization of the PSM and early somites as well as the 3D organization of the surrounding ECM. We then used two-photon live imaging to obtain 3D videos (4D images) of cell dynamics during somite formation. Our results show that PSM cells are highly dynamic, exhibiting constant protrusive activity and cell body movements, and that somite epithelialization involves the progressive organization of these dynamic cells into an aster-like arrangement, a process that takes much longer than the 90 minute interval of each new boundary formation. This reveals and also clarifies the nature and sequence of events in a surprisingly complex morphogenetic process, involving two distinct stages of epithelialization and multiple concurrent cell movements, some of which had not been previously described as contributing to somitogenesis. Perturbation of *de novo* FN matrix assembly does not inhibit the dynamic behavior of PSM cells but impairs cell elongation and alignment, N-cadherin polarization and egression of cells from the somitocoel into the epithelium. These results provide new insights into the complexity of the mesenchyme-to-epithelium transition underlying somitogenesis and demonstrate how a FN matrix is essential to guide dynamic cells into an epithelial structure.

## Materials and Methods

### Embryos and immunohistochemistry

Fertilized chicken eggs were incubated at 38°C until the stages of interest. Embryos and explants were fixed overnight in 4% paraformaldehyde in phosphate buffered saline (PBS) at 4°C, permeabilized with 1% Triton-X100, and incubated overnight with antibodies and dyes diluted in 1% bovine serum albumin (BSA) in PBS. The following antibodies were used: anti-FN (1∶400; Sigma), anti-laminin (1∶100, Sigma), anti N-cadherin (clone 32, 1∶100, BD Biosciences), and the appropriate Alexa Fluor-conjugated secondary antibodies (Molecular Probes). Embryos and explants were treated with ribonuclease A (10 µg/ml, Sigma) and counterstained using ToPro3 (1∶500, Molecular Probes), slowly dehydrated in methanol and cleared with methylsalicylate (Sigma). Somite nomenclature follows that of Pourquié and Tam [36] where the forming somite is defined as somite 0 (s0), formed somites rostral to it are sI, sII and so forth, and prospective somites (PSM caudal to s0) are defined as s-I, s-II and so on.

### Embryo preparation and culture for live-imaging

Stage HH4-5 [37] embryos were microinjected *in ovo* over the anterior primitive streak, with the PCAAGS-GFP vector [38] at a low concentration (0.8–1.4 µg/ml) which, after electroporation, resulted in a mosaic of GFP-positive and GFP-negative cells which facilitated the identification of individual cells within the tissues of interest. An Electro Square Porator ECM830 (BTX Genomics Inc) was used to deliver three to five 25–50 ms 9V pulses, spaced 350 µsec, applying a sharpened tungsten anode under the embryo and a platinum wire cathode above the PSM-prospective territory [39]. After re-incubating overnight, normally developing GFP-expressing embryos were collected with vitelline membranes using a paper ring (Whatman #1) and cleaned in warmed Hanks Balanced Salt Solution (Sigma). Embryos were then mounted ventral-side down over a 0.4 µm pore size Transwell-collagen-coated membrane (Costar, Life Sciences) which was placed on 35 mm diameter #1.5 glass-bottom Petri dishes (World Precision Instruments). The mounted embryo was bathed with Medium 199 (Sigma) with 5% fetal bovine serum and 10% chick serum (culture medium M199) as previously described [40] and the Petri dish placed on a heated stage insert (PECON Tempcontrol 37) inside a custom built microscope incubator box assuring that the embryo, objective and stage were kept at 37–38°C during time-lapse image recording.

### Confocal and two-photon image acquisition

Images of fixed and immunostained embryos and explants were acquired on either a Leica SPE or a Zeiss LSM 510 confocal system. Stacks of optical sections spanning the thickness of the rostral-most PSM and recently formed somites were acquired using a 20x/0.7NA dry or a 40x/1.4NA oil-immersion objectives. 3D images of live embryos were acquired using a Zeiss LSM 510 NLO system coupled to a Ti:Sa IR pulsed laser, pumped by a 5W Verdi laser, and tuned for 880 nm. PSMs of live embryos were scanned dorso-ventrally (approximately 120 µm) using a 20x 0.7NA objective, repeated every 10 minutes for a period of 6–8 hours; bright-field images were acquired simultaneously.

### Inhibition of FN fibrillogenesis

A 70 kDa amino-terminal FN fragment (100 µg/ml; Sigma) was used to inhibit FN matrix assembly [41] as in [33]. For live imaging, embryos were first cultured under the microscope in M199 medium until they formed one somite pair (to ensure normal development) after which the medium was replaced with new warmed M199 containing either the 70 kDa fragment, or BSA (100 µg/ml; Sigma) as a control. In another set of experiments, embryo explants were cultured for 6 hours over a Millipore filter floating on M199 medium [40], containing the 70 kDa fragment (or BSA in controls), after which they were labeled and processed for confocal microscopy as already described.

### Image analysis and quantifications

Z-stacks of confocal and two-photon images were processed and converted using ImageJ (http://rsb.info.nih.gov/ij) and the LOCI's Bio-Formats plugin (http://www.loci.wisc.edu/ome/formats.html), before 3D reconstructions and analysis using Amira v4.1.2 (Visage Imaging, Inc.) and Imaris v5.7.2 (Bitplane, Inc) softwares. The z-depth was rescaled to compensate for refractive index mismatch (by 1.33x in the case of live embryos or by 1.52x in fixed embryos when imaged with dry lenses) and embryo drift was corrected in Amira by 3D reconstructing each time-point 3D stack and repositioning it so that the center of the first formed somite remained stationary.

The Amira software was used to obtain 3D surface reconstructions of PSMs, somites and their somitocoels (by manual segmentation and iso-surfacing of volumes), as well as volume renderings of the 3D distribution of FN, laminin and N-cadherin. 3D coordinates of the somitocoel centroid, basal and apical ends of rostral and caudal somite cells were used to calculate the cell length ( =  Euclidean distance between the cell's apical and basal ends), and centripetal alignment of fusiform epitheloid cells ( =  angle formed between the somitocoel's centroid, and the cell's basal [vertex] and apical ends; Figure S1A). These measurements were done at two time points in each embryo (n = 3; n = 20 cells/embryo [10 rostral and 10 caudal]) and differences between s0 and sII stages as well as between rostral and caudal cells were evaluated using a repeated measures ANOVA. Differences between treatments (*i.e.* control *versus* 70 kDa fragment-treated (n = 3 embryos; n = 20 cells/embryo [10 rostral and 10 caudal])) were evaluated using a nested ANOVA, where the effects on rostral and caudal cells were evaluated separately.

Cell densities in somitocoel and somite epithelium were estimated from the nuclear labeling within the manually segmented volumes (see above) by calculating the average grey intensity *per* µm^3^ and further normalizing to the average intensity of the neural tube at the same axial level. Only somites fully separated from the PSM (≥ sI) were measured and because an ANOVA revealed no significant differences between somites within each treatment, we pooled the different somites to an average *per* explant for each parameter measured, and evaluated differences between treatments (*i.e.* control *versus* 70 kDa fragment-treated) using a *t*-test.

Timing of somite formation and staging were determined by analyzing bright-field image sequences in ImageJ and scoring the appearance of new clefts. Using the 4D images in the Amira software we scored the number of pseudopodia formed by cells of control and 70 kDa fragment-treated embryos and using the Imaris software we manually tracked cell-body movements in 3D, and calculated track lengths and net displacement (distance between initial and final position; Figure S1B) of caudal *versus* rostral cells of control and 70 kDa fragment-treated embryos (n = 3 per treatment; 20 cells per embryo); measurements per treatment were pooled and differences evaluated using a *t*-test. We also determined the fate of PSM cells that were initially either peripheral or in the core and calculated the percentage that became elongated and centripetally aligned (*i.e.* epitheloid) at the end of the video, in control *versus* 70 kDa fragment-treated embryos.

All quantifications are summarized in Table S1. Statistical analysis was performed using JMP!In V4.0 (SAS institute) or Statistica 8.0 (StatSoft).

## Results

### Somite formation involves two stages of epithelialization and contact with a FN matrix

**Figure 1:**
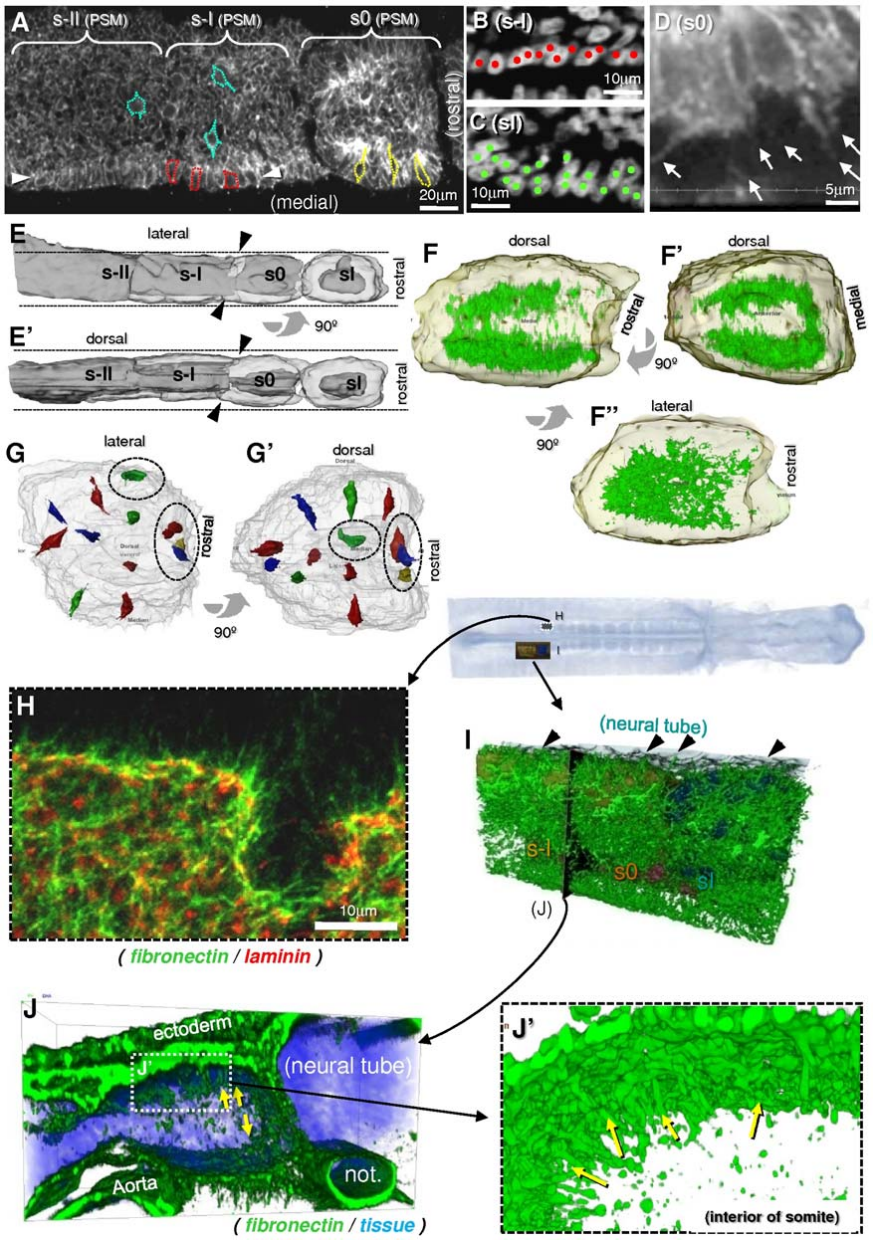
Cell organization, polarization and matrix organization in rostral PSM and epithelial somites (see also Videos S1 and S2). A) Coronal confocal section of chick embryo PSM immunostained for N-cadherin showing s-II, s-I and s0. Different cell shapes can be recognized: mesenchymal polygonal cells (blue), medial cuboidal cells (red) which are aligned forming a cuboidal epitheloid layer (between arrowheads) and elongated spindle-shaped cells (yellow). Red cells in s-I have started elongating and yellow cells in s0 are spindle-shaped. B and C) Coronal confocal sections of a chick embryo PSM stained for DNA showing a detail of the medial epithelium at the level of s-I (B) where cell nuclei are round and basally aligned (red dots), and at level sI (C), where they are oval-shaped and non-aligned basally as in a pseudostratified epithelium (green dots). D) Coronal confocal section of a chick embryo PSM stained for N-cadherin, showing in detail the extension of pseudopodia that connect the PSM cells to their surroundings. E) 3D surface reconstruction of the rostral PSM and sI showing a dorsal (E) and medial view (E′). Transparent surface represents the epithelium and in dark gray the mesenchymal core. The s0 somite is still inserted in the “socket-like” PSM [1], but indentations (arrowheads) reveal where the inter-somitic cleft will soon appear. The rostral and lateral sides of s0 are still mesenchymal. As a result of cell rearrangements between s-II and sI, the PSM narrows medio-laterally and thickens dorso-ventrally (dotted lines in E and E′). F) 3D surface reconstruction (white transparent surface) of s0 somite viewed medially (F), rostrally (F′) and dorsally (F″), showing a volume reconstruction of N-cadherin immunostaining (green) inside. N-cadherin is enriched in the medial, dorsal, ventral and caudal sides, and less so in rostral and lateral sides. Thus the N-cadherin-staining forms a 3D “adhesion basket” in s0. G) 3D surface reconstruction (white transparent surface) of s0 somite viewed dorsally (G) and medially (G′) showing representative cells in the epithelial layer inside, also surface reconstructed (multiple colors). Rostral and lateral cells are elongated but not yet oriented centripetally (dotted circles), while cells in other sides are already aligned. See also Video S1. H) Projection of tangential confocal coronal sections FN-positive fibrils (green) extending away from somite surface, and demonstrating a patchy pattern of laminin immunoreactivity (red). I) 3D volume reconstruction of FN matrix organization (green) surrounding the rostral PSM including s-I and s0 (brown surface) and sI (blue surface). Rostral is to the right and medial to the top. Cables of FN connect the PSM and sI to surrounding tissues (arrowheads). J) Rostral view of FN matrix in a 3D volume reconstructed transversal slab of PSM as shown in I (black plane) at the level of the forming cleft between s-I and s0. Dorsal is upwards, medial is to the right. Cables of FN penetrate inwards into the interior of the somite (yellow arrows), along the lateral surface of epitheloid cells, and between somites, into the nascent cleft. Tissues are represented in light blue. J' is a detail of panel J, showing the cables of FN penetrating the intersomitic cleft. The tissues have been digitally removed to show only the FN matrix (green).

To better understand how cells are organized as somites form, we used N-cadherin immunolabeling and ToPro3 nuclear staining to study cell morphology and polarization in the rostral PSM and newly formed somites. At the level of s-II and s-I, medial peripheral PSM cells are cuboidal (Figure 1A), with round basal nuclei (Figure 1B) and apically polarized N-cadherin (Figure 1A). We define this layer of medial cuboidal cells with the basal side facing outwards as the first stage of somite epithelialization. At the s0 level and further rostrally, medial cells are spindle-shaped (Figure 1A), their nuclei are oval and not aligned basally (Figure 1C), N-cadherin is restricted apically (Figure 1A), and thick basal tapering extensions connect these cells to surrounding tissues (Figure 1D). We define this pseudostratified layer of polarized epithelial cells with basal protrusive activity as the second stage of epithelialization. Other cells in the rostral PSM are polygonal, widely spaced, do not present a noticeable orientation or epithelial organization, and N-cadherin is homogeneously distributed on their surface (Figure 1A).

Eventually, all peripheral cells in a mature somite reach the second stage of epithelialization, but not simultaneously on all sides. In s0, cells in rostral and lateral sides are still non-polarized, unaligned and polygonal, while on all other sides, the peripheral cells have polarized N-cadherin, are spindle-shaped and aligned centripetally (Figure 1A, 1E, 1E′, 1G and 1G′; Video S1). Consequently, a 3D reconstruction of the apical N-cadherin staining reveals a “3D adhesion basket” opened rostrally and laterally (Figure 1F, 1F′ and 1F″).

The PSM is surrounded by an extracellular matrix containing FN [26], [33] and laminin [26]. Laminin immunoreactivity appears as discrete spots distributed homogeneously over the rostral-most PSM and newly formed somite (Figure 1H). This indicates that the laminin matrix is still in early stages of assembly [42]–[44] and only forms a continuous basement membrane later. In contrast, a dense fibrillar FN matrix surrounds the rostral PSM (Figure 1H and 1I; Video S2), and numerous FN fibrils penetrate the tissue (Figure 1J and 1J′). Cables of FN fibrils often align along pseudopodia (data not shown) and are especially frequent at the forming inter-somitic cleft (Figure 1J and 1J′; Video S2), suggesting that cells exert traction forces or use the FN matrix for structural support. We conclude that FN is the likely ECM ligand for the basal protrusions of PSM cells during somite formation.

### The pseudostratified somite epithelium arises through a dynamic process initiated medially

**Figure 2:**
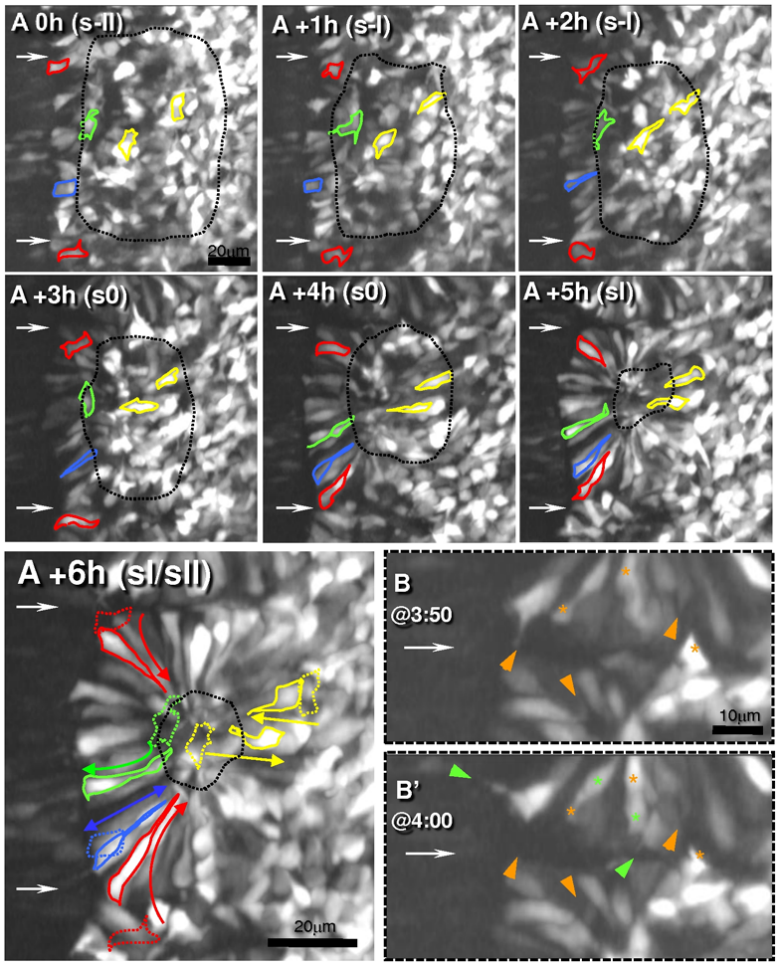
Morphogenetic movements during somite formation in chick embryos (see also Video S3). A) Panels represent seven time-points (spaced 1 hour) of a 4D two-photon imaging sequence (each is a 3D reconstructed 30 µm coronal “slab” through the somite's equator (rostral is to the top, medial to the left) of embryos expressing a mosaic of GFP-positive and negative cells. Thin horizontal arrows point to areas where intersomitic boundaries form. Five cells were traced (outlined in color) to depict different morphogenetic movements: *i*) Cuboidal medial cells elongate and become spindle-shaped (blue). *ii*) Some cells are recruited to the medial epithelium via accretion (red) and undergo a change in shape and orientation to conform to the orientation of medial elongated cells. *iii*) Lateral PSM cells and core mesenchymal cells (both yellow) converge and elongate. *iv*) Core cells egress (green) into the epithelium, becoming spindle-shaped and centripetally aligned. In the last panel (A+6h) the initial positions of the cells (A+0h) are drawn with dotted lines, and colored arrows represent the overall morphogenetic movements. The size of the mesenchymal region, *i.e.* the somitocoel (black dotted lines) becomes progressively smaller during these 6 hours. B) Detailed view of time-points 3:50 (B) and 4:00 (B′) showing pseudopodia retracting (orange arrowheads) and new ones forming (green arrowheads), similar to the tapering extension observed in fixed embryos (see Figure 1D and last segment of Video S3). Furthermore, massive cytoplasm movements inside cells are also visible (asterisks; where green marks cytoplasm translocations; see also Video S3). These movements occurred throughout the image recording period indicating that the dynamic behavior was retained even after cells became epitheloid.

The data from fixed embryos showed that the mesenchyme-to-epithelium transition underlying somite formation appears to occur progressively around the somite. To analyze this phenomenon *in vivo*, we obtained 4D images centered on PSM level s-II over time, until this area assembled as a new somite and reached sII (Figure 2; Video S3). Interestingly, during the whole period, cells displayed highly dynamic protrusive and motile activities (Figure 2B and 2B′), even after becoming incorporated into the epithelium (Video S3 and Table S1). Close analysis of the videos allowed us to discriminate a number of different morphogenetic movements underlying somite epithelialization: *i*) Medial cuboidal cells elongate and become spindle-shaped (Figure 2A and Video S3, blue cell). *ii*) Mesenchymal cells adjacent to these medial spindle-shaped cells attach “one-by-one” to their basal ends and “zip” along their surface, becoming elongated and centripetally aligned (Figure 2A and Video S3, red cells), a process best described as cell accretion. Accretion first spreads to the ventral and dorsal PSM side, then to the caudal side of s0 (compare Figure 2A+3h with 2A+4h), only reaching the rostral side in late s0/sI (compare Figure 2A+4h with 2A+5h). *iii*) Epithelialization of the lateral side occurs last (≥sI; compare Figure 2A+5h with 2A+6h) and involves yet another mechanism: cells from the lateral-most PSM and the core elongate, intercalate and condense into an epithelium (Figure 2A and Video S3, yellow cells). This movement seems to be responsible for the medio-lateral narrowing and dorso-ventral thickening of the prospective somite and its detachment from the intermediate mesoderm (Figure 1E and E′). Throughout the image recording period we also observed a fourth type of morphogenetic movement: *iv*) Egression of cells from the center of the PSM/somites (Figure 2A and Video S3, green cell) outwards into the forming epithelium. Ingression from the epithelium to the core also occurred, but was less frequent, hence the somitocoel became progressively smaller (Figure 2, black dotted contours). Core cells egress by extending protrusions which penetrate the apical cell-cell adhesions, and then undergoing amoeboid movements that translocate the cell body into the epithelium where cells become spindle-shaped and centripetally aligned (Figure 2A and Video S3 green cell). Furthermore, *v*) we recorded cells “jumping” from one prospective somite to another as previously described by [1]. The fact that cells freely switch between periphery and core, and between adjacent somite territories suggests that either cell fate choices are flexible, or that differently committed cells sort according to their fate.

We conclude that somite formation is a complex process involving a combination of multiple morphogenetic movements that occur continuously over a period of at least 6 hours, without noticeable periodic changes of cell behavior every 90 minutes. Furthermore, the somitic epithelium is clearly not a “conventional” epithelium, as its cells retain many characteristics of mesenchymal cells. Hence, we will refer to these cells as epitheloid rather than epithelial cells.

### Inhibition of FN matrix assembly impairs PSM cell elongation and alignment without affecting cell dynamics

We have previously shown that somitogenesis is impaired when embryo explants are cultured with the 70 kDa N-terminal FN fragment [33], known to inhibit the incorporation and assembly of endogenous FN molecules into fibrils [41]. As our live imaging data demonstrates that somitogenesis involves extensive cell movements and basal protrusive activity towards the surrounding FN matrix, we analyzed the effect of perturbing FN matrix assembly on PSM cell behavior.

**Figure 3:**
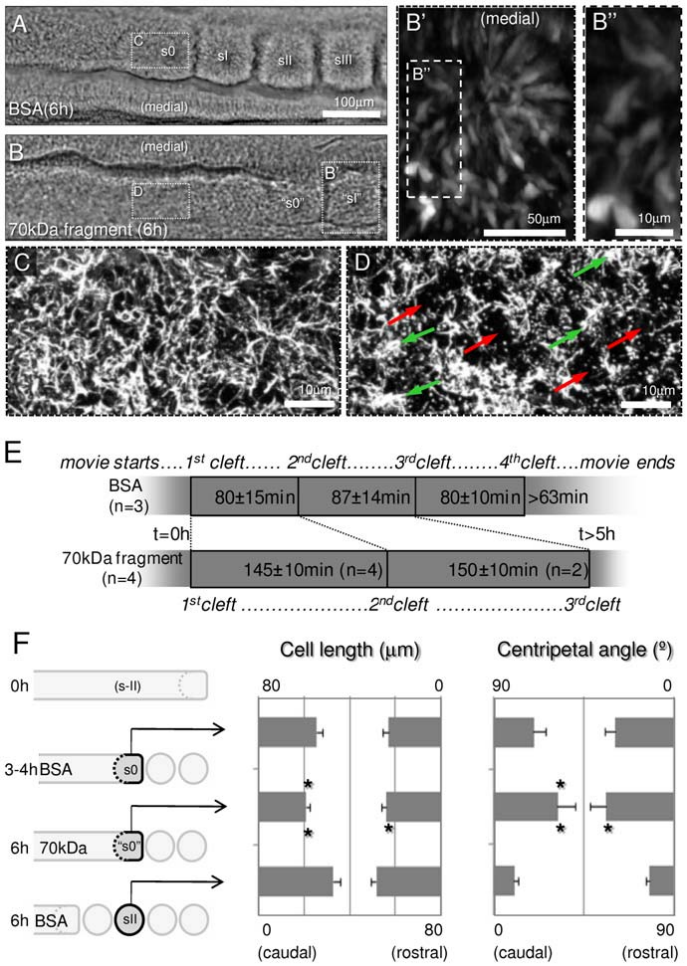
Inhibiting FN matrix assembly impairs somite formation by affecting cell elongation and alignment. A and B) Bright-field images showing equivalent halves of embryos cultured for 6 hours either under control (BSA) conditions (A) or with the 70 kDa FN fragment (B). The control embryo formed three complete somites (sI-sIII) and an advanced s0 (A), while the 70 kDa fragment-treated embryo formed only one somite (marked “sI”) and an incipient “s0” (B). B′ is a two-photon section showing an “sI” of a GFP-electroporated and 70 kDa fragment-treated embryo showing impaired cell elongation and alignment (see also Video S4), particularly in the caudal side (B″; compare with Figure 2A+6h). C and D) FN matrix covering the dorsal surface of the PSM at equivalent axial positions of control explants (s0, C) and explants treated with the 70kDa fragment (“s-II”, D). Ectoderm-associated FN was digitally removed to show only the PSM FN matrix. Explants treated with the 70 kDa fragment (D) show numerous large holes in the FN matrix (red arrows) interspersed with dense agglomerates of FN (green arrows), contrasting with the more uniform fibrillar matrix of control explants (C). E) Timing of intersomitic cleft formation as determined from analysis of bright-field time-lapse image sequences. Control embryos (n = 3) formed three new somites in approximately 4 hours, while experimental embryos (n = 4) took almost 5 hours to form either one (n = 2) or two (n = 2) new somites. F) Graphical representation of cell lengths and centripetal angles of rostral (n = 10 cells/embryo) *versus* caudal (n = 10 cells/embryo) somitic cells in control embryos at mid-culture (s0, n = 3), experimental embryos at 6 hours (“s0”, n = 3) and control embryos at 6 hours (sII, n = 3). Comparing cells in “s0” with the equivalent cells of control embryos, which had matured to sII stage, showed that caudal cells were significantly less elongated and aligned (*P*<0.001) in the “s0”, and so were rostral cells (*P* = 0.004 and *P*<0.001, respectively). Comparing cells in “s0” to cells of control s0 revealed significant differences in caudal cell elongation (*P*<0.001) and alignment (*P* = 0.007) whereas no differences in those parameters were detected rostrally (*P* = 0.643 and *P* = 0.368, respectively). Error bars represent 95% confidence intervals. Asterisks represent significant differences of “s0” when compared to sII (bottom asterisk) or s0 (upper asterisk).

Embryos cultured in medium with BSA (control, Figure 3A) form three or four somites in 6 hours, and an even fibrillar network of FN matrix can be observed (Figure 3C). In contrast, embryos cultured with the 70 kDa FN fragment (Figure 3B) form only one or two somites and the FN matrix has numerous large holes interspersed with dense agglomerates of FN (Figure 3D). Analysis of bright-field image sequences of live-imaged control embryos (n = 3) showed that new somitic clefts form every 80–87 minutes (Figure 3A and 3E), as expected. In contrast, fragment-treated embryos (n = 4) took 145 minutes to form the first somite pair and only two out of four embryos gave rise to a second somite pair, which formed in 150 minutes (Figure 3B and 3E). Thus the disruption of the FN matrix first delays and then effectively halts the formation of new somites.

Analysis of cell movements in 4D images of treated embryos showed that, despite the perturbation of the FN matrix, the medial wall of cuboidal epitheloid cells forms and the movements of accretion, egression and condensation still occur (Video S4). In control and fragment-treated embryos (n = 3; 20 cells/embryo each) cell bodies moved an average distance of 39.6 µm/37.6 µm every 90 minutes, for a net displacement of only 4.8 µm/5.5 µm, respectively (Table S2). This demonstrates the dynamic nature of cells within the rostral PSM and shows that this dynamic behavior is not affected when FN matrix assembly is perturbed (*P* = 0.1468 and *P* = 0.1582, respectively). Although the 4D images do not permit resolution of fine filopodial protrusions, we scored the formation of pseudopodia and found that embryos treated with the 70 kDa fragment formed the same number of pseudopodia as control embryos (Table S1) showing that the dynamic behavior is preserved. Despite this, the PSM cells of the fragment-treated embryos never formed the expected aster-like arrangement of control somites (compare Figure 2A+6h with Figure 3B′), most likely due to a defect in cell organization (Figure 3B″).

To test this, we measured the alignment and lengths of PSM/somitic cells. In control embryos, cells that were at s-II level at time 0 h had been incorporated in sII after 6 hours of culture (Figure 3A), while in fragment-treated embryos (n = 3) these cells only reached a s0-like organization which we designate “s0” (Figure 3B and 3F). Notably, both rostral and caudal cells from control sII are significantly more elongated and aligned than the equivalent cells from the “s0” (Figure 3F). Thus the FN matrix plays a crucial role in cell elongation and alignment during somitogenesis.

We next assessed whether the elongation and alignment of cells in “s0” is equivalent to an s0. Normally, in a forming somite, the caudal side epithelializes before the rostral one (Figure 1; [45]. Comparing cell elongation and alignment in s0 *versus* “s0” reveals no differences rostrally, but caudal “s0” cells are significantly less elongated and less aligned than in s0 (Figure 3F). This demonstrates that “s0” somites are not just delayed, but that the epithelialization of their caudal walls also is greatly impaired.

We conclude that an intact FN matrix is not essential for the pseudopodial or motile behavior of PSM cells, but is required to orient their dynamic behavior and guide them into an aster-like arrangement.

### FN matrix plays a crucial role in N-cadherin polarization during somitogenesis

**Figure 4:**
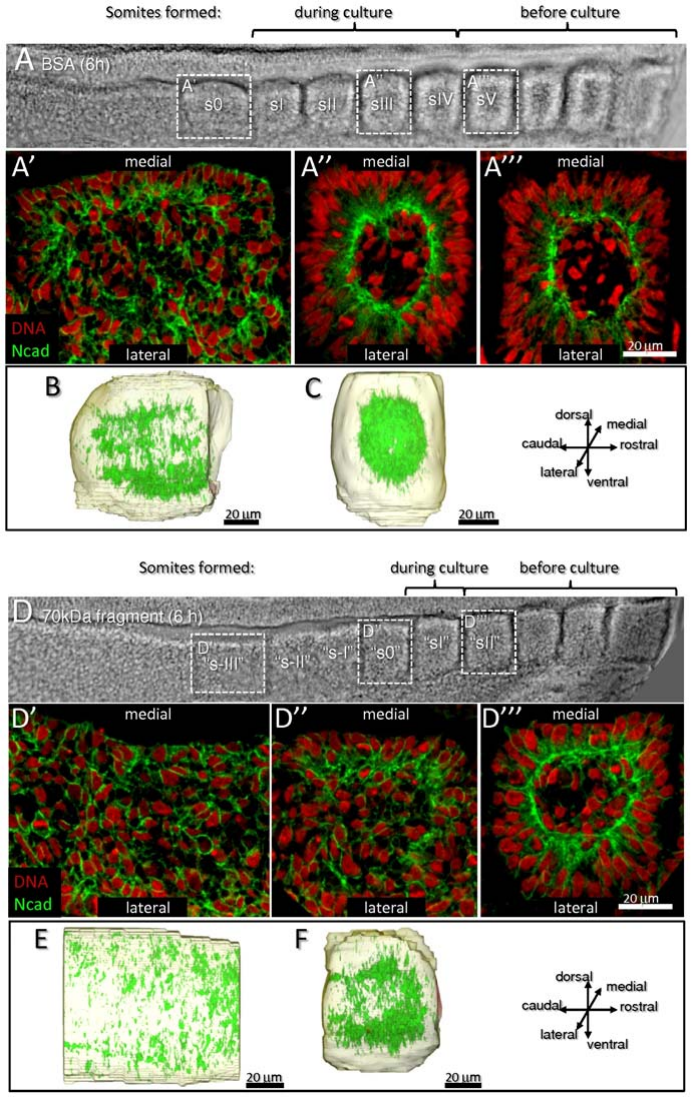
N-cadherin fails to polarize apically when FN fibrillogenesis is inhibited. Bright field (A, D), coronal confocal sections (A′, A″, A″′, D′, D″, D′″) of control (A) and 70 kDa FN fragment-treated (D) explants cultured for 6 hours, immunostained for N-cadherin (green) and labeled for DNA (red) and 3D surface reconstruction with N-cadherin labeling (green in B,C,E,F) of selected volumes. A) Control explants form four new somites. In s0 (A′), medial cells are starting to elongate and to polarize their N-cadherin apically. In sIII (A″) and sV (A″′), cells have become centripetally aligned, with oval-shaped nuclei and apically restricted N-cadherin immunoreactivity. B–C) Lateral view of N-cadherin labeling shows a “3D adhesion basket” in s0 (B). By sIII (C) the N-cadherin labeling has become more apically restricted and has closed rostrally and laterally thus forming a ball. D) 70 kDa fragment-treated explants form one or two somites; the depicted embryo formed one (“sI”) and has an advanced “s0”. The tissue at the same axial level as A′ (“s-III”, D′) shows no sign of epithelialization or cell elongation. Medial cells in the forming somites of 70kDa fragment-treated explants (“s0”, D″; axially equivalent to A″) show incipient elongation and N-cadherin polarization. Cells of somites that had formed before culture (“sII”, D″′) are less polarized and less elongated than cells at the equivalent axial level in control explants (sV). E–F) Lateral view of N-cadherin labeling in 70kDa fragment-treated explants show dispersed N-cadherin localization in the “s-III” (axial equivalent to B). The “s0” (axial equivalent to C) depicts a more intense labeling in the rostral portion, resulting in an “adhesion basket” that is opened caudally.

In order to assess whether perturbing the FN matrix also affects the apical polarization of N-cadherin, embryo explants were cultured for 6 hours with BSA (n = 4; Figure 4A) or the 70 kDa FN fragment (n = 6; Figure 4D) and immunostained for N-cadherin. Analysis of our results showed that in control explants, s0 cells have started to polarize their N-cadherin apically (Figure 4A′) and sIII cells are elongated, aligned and have apically polarized N-cadherin (Figure 4A′). In contrast, in fragment-treated explants (n = 6; Figure 4D), the axial equivalent to s0 of controls have an “s-III” morphology and do not show any sign of epithelialization (Figure 4D′). The axial equivalent to control sIII cells have only reached an “s0” morphology with cuboidal medial cells and present only a slight apical enrichment of N-cadherin (Figure 4D″). 3D reconstruction of the N-cadherin labeling further confirms the differences between control and 70 kDa fragment-treated explants. The s0 in control embryos has a “3D adhesion basket” opened rostrally and laterally (Figure 4B) and in sII it has formed a ball (Figure 4C). In contrast, the two equivalent tissues in fragment-treated explants have no obvious apical polarization of N-cadherin (“s-II”; Figure 4E) or have a defective “3D adhesion” basket with the caudal side opened (“s0”; Figure 4F). Thus in addition to cell elongation and alignment abnormalities (Figure 3F), the cells of fragment-treated explants also show impaired N-cadherin polarization which in “s0” is most severe caudally.

We further noticed that perturbing the FN matrix also affects somites that were already formed before culture. In control explants, sI cells progressed to sV during the 6 hour culture and were elongated, centripetally aligned with apically restricted N-cadherin (Figure 4A″′). Cells at sI level in 70 kDa fragment-treated explants, did not develop into a typical sV (Figure 4A″′), but remain less elongated and display less polarized N-cadherin immunoreactivity (Figure 4D″′). In fact, these cells were even less elongated and less polarized than cells in a control sIII (Figure 4A″). This shows that the FN matrix not only promotes cell elongation and polarization during the formation of new somites, but is also crucial to maintain the epithelial state of already formed somites.

### Egression of core cells into the somite epithelium requires an intact FN matrix

**Figure 5:**
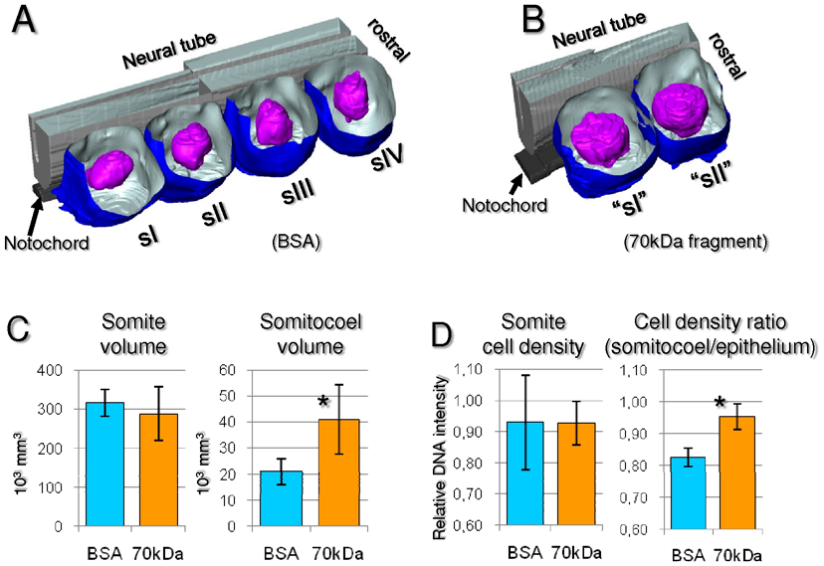
Inhibition of FN fibrillogenesis impairs egression of cells from the somitocoel to the somite epithelium. A–B) Representative examples of 3D surface reconstructions of somites (blue; dorsal cap digitally removed) and their somitocoels (purple) formed in embryo explants at the end of 6 hours of culture with BSA (n = 4; A) or with 70 kDa fragment (n = 6; B). Three of the six explants treated with the fragment formed two somites as depicted in B; the remaining ones formed only one somite. C) Quantification of somite and somitocoel volumes and cell densities (fluorescence intensity of DNA labeling). Only somites fully separated from the PSM (≥ sI) were measured. Since an ANOVA revealed no significant differences between somites formed during the 6 hour culture period within each treatment, we pooled the measurements of the different somites to an average *per* explant (C). There is no significant difference (*P* = 0.129) in somite volume between control and fragment-treated explants, but somitocoels of fragment-treated explants are significantly larger (*P*<0.0001). D) Quantification of cell density in the whole somites shows no difference (*P* = 0.990) between control and 70 kDa fragment-treated explants. However, the cell density ratio (somitocoel/epithelial portion) is significantly higher (*P* = 0.043) in somites of 70 kDa fragment-treated explants. Bars represent 95% confidence intervals.

We noticed that in embryos cultured with the 70 kDa fragment, somites have unusually large somitocoels, suggesting a possible defect in the recruitment of core cells into the epithelium. To address this, we started by tracking individual cells in the 4D images. In control embryos (n = 3), 95% of tracked core cells (n = 35/37 cells) from s-II became epitheloid after 6–8 hours, while in experimental embryos (n = 3) only 29% of tracked core cells (n = 10/35 cells) became part of the epithelium (Table S1), suggesting a defect in the egression of cells from the core to the periphery. To further quantify this phenomenon, we measured the volumes of somites formed and their somitocoels during a 6 hour culture period in control (n = 4) and 70 kDa fragment-treated (n = 6) explants (Figure 5A and 5B). Total volume of somites does not differ significantly, but in 70 kDa fragment-treated explants the somitocoels are significantly larger, representing 13.2% of the total volume of the somite, in contrast to only 6.5% in control explants (Figure 5C; Table S1). Furthermore, in control embryos, somitocoel cell density is lower than that of the surrounding epithelium while in fragment-treated embryos it is as high as in the epithelium (Figure 5D). This strongly suggests that the ability of core cells to egress into, and become part of the somitic epithelium is seriously hampered when FN fibrillogenesis is inhibited.

## Discussion

### Somite epithelialization is a complex and continuous event

The chick PSM segments every 90 minutes, giving rise to a new somite. This periodicity is achieved through a molecular segmentation clock, evidenced by oscillations of gene expression in the PSM, which is the hallmark of somitogenesis [23], [24]. Here we used 3D live imaging of the full chick PSM in a mosaic of GFP-labeled and non-labeled cells to study the cellular transformations involved in somite formation. Surprisingly, our time-lapse images revealed that the epithelialization of the forming somite does not occur in a periodic fashion every 90 minutes. Rather, a continuous series of events, spanning a period of at least 6 hours, bring about the assembly of cells into the epithelial somite, which only becomes complete as it reaches stage sII.

Our data show that somitogenesis involves two distinct epithelialization events. Although it has previously been reported that the chick PSM shows signs of precocious epithelialization [18], [46], [47] and that the somitic epithelium is pseudostratified [19], [21], it has gone largely unnoticed that these two epithelia are morphologically distinct and that the cuboidal epithelium of the rostral PSM is characteristic of its medial cells.

**Figure 6:**
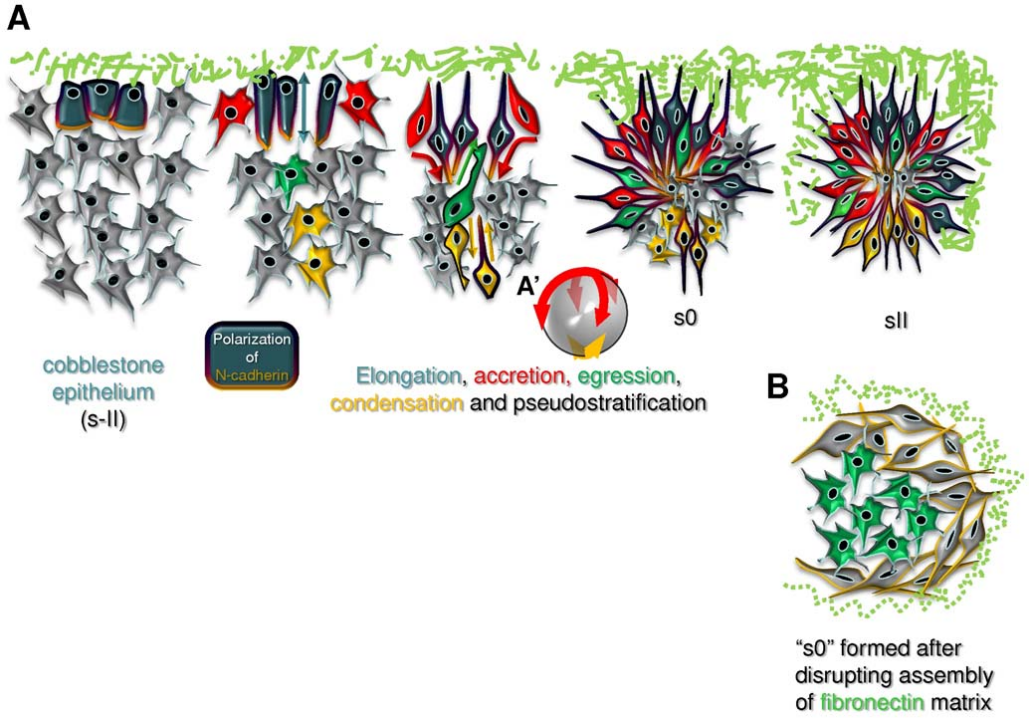
Model of morphological somite formation and morphogenetic movements. A) Schematic representation of a “hypothetical” coronal slice representing stages of somite formation. Medial is up and rostral to the right. The different morphogenetic movements that contribute to the assembly of the somite epithelium are represented: Medial cells first become cuboidal with basal nuclei (dark green cells), then these cells elongate and recruit other cells to the epitheloid layer via accretion (red cells); accretion spreads ventrally and dorsally (not shown), and eventually to the caudal and rostral side. Simultaneously, cells egress from the core into the epitheloid layer (green cells), and, finally, at the lateral side of the somite, cells elongate, intercalate and condense (yellow cells). In s0, the rostral and lateral sides epithelialization is only completed after the somite separates from the PSM. The FN matrix is depicted in green. A′ depicts how the accretion and condensation “spread” through the whole somite to complete the “spherical” ball of epitheloid cells. B) When the assembly of the FN matrix is disrupted, PSM cells (especially caudal cells) do not polarize or orient centripetally and core cells fail to egress into the epithelium.

The transformation of the mesenchymal chick PSM into epitheloid cells starts medially with the formation of the cuboidal epithelium. These cells later elongate into a pseudostratified arrangement and while doing so, they recruit adjacent cells by accretion (Figure 6A). It has been shown that medial PSM cells form somites autonomously, whereas lateral PSM cells require the presence of medial ones [48]. By showing that both steps of epithelialization start medially and that these medial cells recruit adjacent cells into the forming epithelium, our images provide evidence that reinforces the idea that medial cells have an organizing function during somitogenesis. Although accretion has not previously been identified for somitogenesis, it is known to occur, for example during notochord development [49], [50] and primordial germ cell cluster formation [51].

The lateral side of the chick somite epithelializes last when lateral mesenchymal cells rearrange by intercalating and elongating into a centripetal arrangement (Figure 6A), reminiscent of the convergence and extension movements of dorsal mesoderm cells in amphibian embryos [52].

Finally, egression of cells from the forming somitocoel is an unexpectedly large contributor to the somitic epithelium (Figure 6A). In fact, core cells are as likely to end up in the peripheral epitheloid layer as peripheral cells. The observation that an epithelium can assemble from mesenchymal cells egressing into it from the apical side and aligning along the existing epitheloid cells is unexpected and few other examples exist [53]. High-resolution time-lapse imaging of situations where an epithelium assembles from a group of mesenchymal cells (e.g. vasculogenesis; condensation of the metanephric mesenchyme during kidney development) should clarify whether this mechanism is a general feature of mesenchyme-to-epithelium transitions.

The assembly of the somitic epithelium is a dynamic process and the dynamic behavior of its cells continues after the somite forms. The constant translocations of cell bodies within the epitheloid layer may be a response to continuous cell recruitment into this epithelium through egression, which gives it the pseudostratified appearance. Pseudostratification seems to be typical of embryonic epithelia which rest on FN-rich and laminin-sparse matrices [8] as is the case of rostral PSM and early somites. We conclude that the somitic epithelium is clearly not a conventional epithelium, neither morphologically nor behaviorally, as its cells retain several mesenchymal characteristics. Future live imaging studies should address whether pseudostratification in embryonic epithelia represents a structural organization *per se* or whether it simply reflects that these embryonic epitheloid tissues are formed by cells which are highly dynamic and in transit (as is the case of somite cells which later disperse and move on to give rise to different tissues of the adult organism).

### The FN matrix orients PSM cells into an aster-like somite conformation

Our previous studies revealed that the FN matrix of the PSM is the product of a collaboration between ectoderm and PSM, where ectoderm provides the bulk of the FN protein, and the PSM cells assemble this FN into a fibrillar matrix [33]. Furthermore, the 70 kDa FN fragment inhibits somitogenesis [33] by halting *de novo* FN fibrillogenesis [41], [54], [55]. Here we show that culture of embryo explants in the presence of the 70 kDa FN fragment for 6 hours causes a disruption in the FN matrix, as evidenced by large holes and areas with apparently collapsed fibrils. A similar effect is seen in cultured cells where an already assembled matrix is progressively lost if FN molecules are not continuously added to it [56]. Thus, young FN matrices are in a constant turnover and inhibiting FN matrix assembly gradually leads to a net loss of matrix [56]. Observing cell behavior in 3D time-lapse images of live embryos cultured in the presence of the 70 kDa FN fragment gave us the unique opportunity to determine what aspects of somitogenesis fail as the surrounding FN matrix weakens over time.

The dynamic behavior of PSM cells suggests that they are continuously sensing and adjusting to the surroundings (other tissues and the ECM). Our results show that the inhibition of FN matrix assembly does not affect PSM cell movements and pseudopodial activity, but perturbs N-cadherin polarization, cell elongation, centripetal alignment and egression (Figure 6B). This suggests that the FN matrix normally serves as a cue that orients the dynamic behavior of PSM cells, polarizes the cell-cell adhesions to the apical side and serves as a basal anchoring point essential for cell elongation and alignment. FN matrices are crucial in inducing cell elongation and polarization in young embryonic epithelia before they mature and assemble their laminin-containing basement membranes [57]–[59]. Furthermore, FN matrices also induce cell elongation and orient protrusive activity during dorsal mesoderm development in *Xenopus* [60]. Many studies have also provided evidence of cross-talk between FN signaling and cadherins [16]. For example, a basal FN matrix polarizes N-cadherin to the apical domain of zebrafish myocardial cells [58] and downregulates E-cadherin basally in cleft cells of branching epithelia [57]. Furthermore, in *Xenopus* mesoderm, β1 integrin signaling modulates C-cadherin adhesiveness to the “correct” level to permit tissue rearrangements [61]. In fact, the presence of FN around the rostral PSM correlates with an increase in cell-cell adhesion between PSM cells [62]–[64]. Thus a FN matrix is important both as a basal orienting cue and in polarizing cell-cell adhesion to the apical side in several developing systems. These basal and apical attachments are essential for creating and maintaining the tensile strength that supports cell elongation and cell body movements, and in turn may also modulate the cytoskeleton and intracellular signaling pathways thereby controlling cell shape and differentiation [7]. Moreover, we also saw a loss of N-cadherin polarization and cell elongation in somites that were already formed before the addition of the 70 kDa FN fragment. Therefore, cues from a basal FN matrix seem to be crucial, not only to induce, but also to maintain somite cell polarization and elongation.

Egression is significantly impaired when FN matrix assembly is perturbed, demonstrating that an intact FN matrix is required to support and direct cell egression. FN matrices are flexible and elastic and are extensively modifiable by cell traction forces [65]–[67]. The presence of cables of FN penetrating the somite epithelium indicates that somitocoel cells may pull on the matrix to bring themselves into their final position. We propose that without the presence of a strong and continuous matrix, many cells fail to egress and remain in the somitocoel, resulting in fewer cells in the epithelium.

### The FN matrix aids somitic cleft formation

It has previously been hypothesized that somite epithelialization starts at the rostral border of s0 continuing in the caudal direction and finishing at the caudal border as it separates from the PSM [1]. However, more recent observations suggested that, in the chick embryo, the caudal part of s0 epithelializes first [45], [68]. Our analysis of cell shape and orientation, cell movements and the organization of the N-cadherin “3D adhesion basket” in s0 provide a formal demonstration that the caudal wall indeed epithelializes well before the rostral one (Figure 6A).

Work in recent years has established that cleft formation involves a cross-talk between cells caudal and rostral to the forming cleft, whereby cells caudal to the prospective boundary (rostral side of s-I) instruct cells rostral to the boundary (caudal side of s0) to epithelialize [68]. The transcription factor *Mesp2/Meso-1* expressed in the cells caudal to the prospective boundary upregulates *EphA4* expression in these cells [46], [69]. Subsequent binding between EphA4 and EphrinB2 present on the cells rostral to the boundary, induces a signaling event that downregulates the activity of the GTPase Cdc42, leading to cell epithelialization [69]. Interestingly, these authors demonstrate that EphrinB2 signaling not only downregulates Cdc42 activity, but also acts through yet another unknown mechanism to promote cleft formation. Here we show that the inhibition of FN fibrillogenesis dramatically affects the caudal side of the somite, indicating that FN plays a crucial role in its epithelialization. EphrinB1 and EphrinB2 reverse signaling have been shown to increase the affinity of β1 integrins for FN, stimulating cell attachment and motility on a FN matrix [70], [71]. Thus, during somite boundary formation, EphrinB2 signaling may increase integrin engagement to the FN matrix in a cell autonomous manner. Consequently, accreting caudal s0 cells would bring the external FN matrix into the forming cleft with them, a process also seen during cleft formation in salivary gland branching morphogenesis [4]. Newly synthesized FN is then assembled to fill in the space between the gland bud surface and the cells at the leading edge of the cleft, thus stabilizing the cleft further [4]. *Itga5* mRNA is expressed in newly formed somites and *Fn1* mRNA is expressed in their caudal half [33], indicating that newly assembled FN may play the same role in the somitic clefts. Therefore we propose that EphrinB2 signaling would promote epithelialization in two complementary ways: (1) by lowering the activity of Cdc42 promoting epithelialization [69] and (2) by stimulating the binding of caudal s0 cells to the FN matrix which, in turn, induces N-cadherin polarization, cell elongation and centripetal alignment. In agreement with this, *Ephrin-B2a* and *Fn1* have been shown to collaborate in somite cleft formation and maintenance in zebrafish [29].

### Conclusion

Here we present data indicating that during chick somite formation the FN matrix acts as a fibrillar network that spatially orients the highly dynamic mesenchymal PSM cells, bringing them into the aster-like arrangement of spindle-shaped cells characteristic of the epithelial somite. We propose that it does so by inducing the polarization of N-cadherin to the apical domain of PSM cells, thus reinforcing cell-cell adhesions, and by serving as a basal anchoring scaffold, giving PSM cells a second point of attachment essential for acquiring the tensile strength necessary for elongation and orientation in 3D. We further propose that when EphrinB2 reverse signaling induces the somitic cleft, caudal s0 cells adhere strongly to the FN matrix, accreting cells pull the superficial FN matrix into the cleft and then new somite-derived FN is assembled to fill in the cleft, stabilizing it. Finally, it is tempting to speculate that the progressive accumulation of FN in the cleft promotes the alignment and polarization of the cells in the rostral side of the following somite. In this scenario, a fibrillar FN network is absolutely crucial to package cells into a somite.

## Supporting Information

Figure S1 Cell shape and movement measurements. A) Diagram representing the cell's length (distance a-b) and centripetal alignment angle (α; note that the smaller the angle, the better aligned a cell is). “a” represents cell's apical end, “b” the cell's basal end, and “c” is the somitocoel's centroid. See materials and methods for more details. B) 3D reconstruction of tracks of cells whose cell body movement was used to the calculate full track length (magenta line) and net cell body displacement (white vector). Rostral is to the right and lateral to the top. Bright-yellow spheres represent the position of the cell bodies of tracked cells in the last time-point. GFP-expressing cells are 3D reconstructed in transparent light green.
(1.35 MB TIF)

Table S1 Effect of inhibiting FN matrix assembly on PSM cell protrusive and motile activity, elongation and alignment, and probability of egressing. Values presented are averages ±95% confidence intervals.
(0.07 MB DOC)

Video S1 3D surface reconstruction of an s0 stage somite and of cells in the epithelial layer showing their typical shape and orientation. The somite is oriented rostral towards the right, dorsal upwards. Cells in rostral wall are not centripetally oriented. During the second 3D rotation, the surface of reconstructed cells changes color to show apical accumulation of N-cadherin (green). Cells that are well oriented have most N-cadherin accumulated on the apical end, i.e. towards the interior of the somite. For more details refer to Figure 1 of the manuscript.
(5.50 MB MOV)

Video S2 Organization of the FN matrix in the nascent somite. The video is an animation showing all sides of the 3D reconstruction of fibronectin (green) matrix organized around somite sI (blue), s0 (dark brown) and s-I (light brown). Rostral is to the right. The “outward cables” of FN are clearly seen radiating from the surface of the somites in formation. In the middle of the Video only s0 is depicted, to show how the fibronectin matrix is organized on all sides; the medial wall is less well covered with fibronectin than the dorsal and ventral walls. The caudal and cranial walls of s0 are not yet covered with FN. In the last segment of the video, the surface of the PSM and sI somite are removed to show only the FN, and the “inward cables” penetrating the PSM, especially in the prospective cleft. For more details refer to Figure 1.
(10.22 MB MOV)

Video S3 Multiphoton 4D image sequence of GFP expressing-cells in chick embryo PSM showing cell movements during somite formation. First segment of the video shows a portion of the PSM of a chick embryo (rostral is upwards, medial is to the left). In the beginning only one somite is formed, but at the end of 6 hours four new somites have formed. After zooming into a single somite the details of individual cell movements are shown. Several different morphogenetic stereotypical movements are identified in cells with different colors. Blue represents elongation of a medial cell, red cell accretion in the rostral and caudal walls, green egression, and yellow mesenchymal elongation and intercalation in the lateral wall. In the end, a zoom of a forming intersomitic cleft shows the continuous extension and retraction of pseudopodia. For more details refer to Figure 2 of the manuscript.
(8.03 MB MOV)

Video S4 Effect of treatment with 70kDa FN fragment on rate of somite formation and cell movements The video starts by showing two side-by-side bright-field image sequences of a control (BSA; upper panel) and 70kDa treated embryo (bottom panel), to compare rates of somite formation. FN matrix assembly disruption slows down and eventually halts somite formation and prevents cells from organizing into an aster of spindle-shaped centripetally aligned cells. In the last segment of the Video formation of pseudopodia is shown in “freezed-frames”, demonstrating that pseudopodial activity is not affected by the inhibition of FN fibrillogenesis. For more details refer to Figure 3 of the manuscript.
(9.37 MB MOV)
