# Supplementary figures and images for: Dynamic 3D Cell Rearrangements Guided by a Fibronectin Matrix Underlie Somitogenesis
Source: PLoS One. 2009 Oct 15;4(10):e7429. doi: 10.1371/journal.pone.0007429 (PMC2759537; doi:10.1371/journal.pone.0007429)

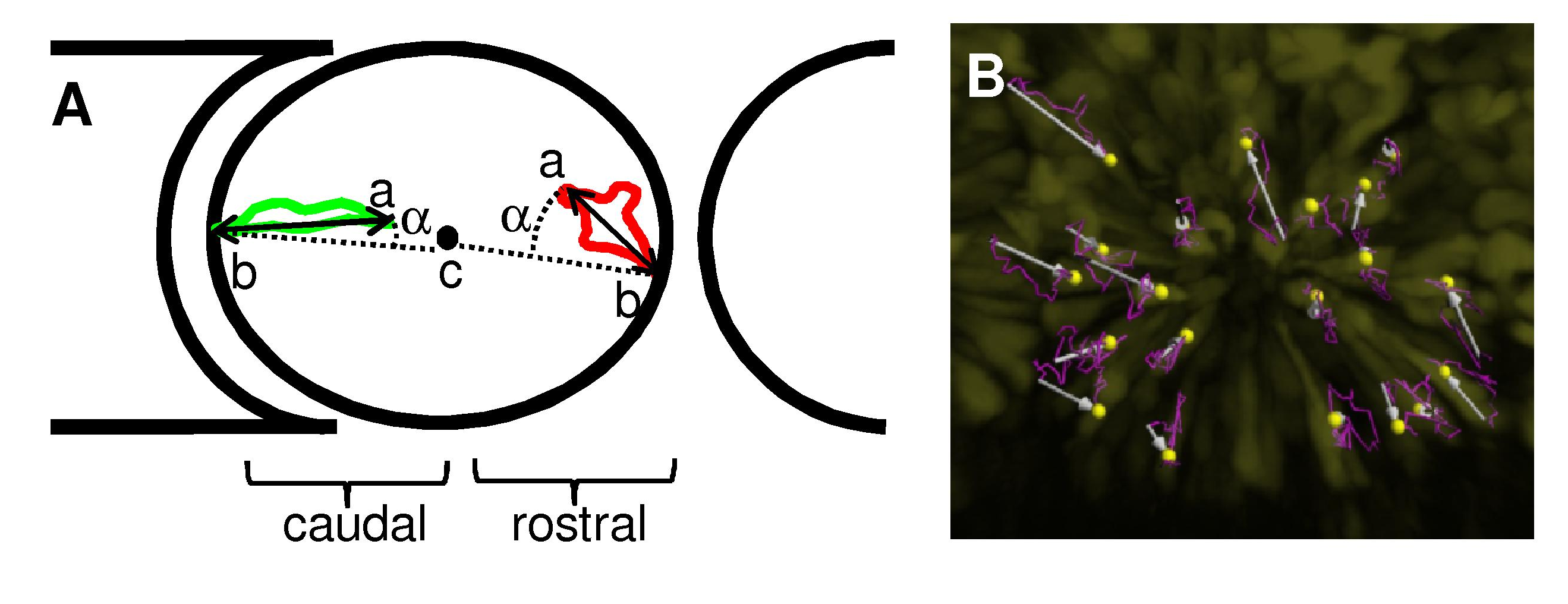

Supplement: Figure S1 — Cell shape and movement measurements. A) Diagram representing the cell's length (distance a-b) and centripetal alignment angle (α; note that the smaller the angle, the better aligned a cell is). “a” represents cell's apical end, “b” the cell's basal end, and “c” is the somitocoel's centroid. See materials and methods for more details. B) 3D reconstruction of tracks of cells whose cell body movement was used to the calculate full track length (magenta line) and net cell body displacement (white vector). Rostral is to the right and lateral to the top. Bright-yellow spheres represent the position of the cell bodies of tracked cells in the last time-point. GFP-expressing cells are 3D reconstructed in transparent light green. (1.35 MB TIF) [file pone.0007429.s001.tif]
