# Supplementary material for: Dynamic 3D Cell Rearrangements Guided by a Fibronectin Matrix Underlie Somitogenesis
Source: PLoS One. 2009 Oct 15;4(10):e7429. doi: 10.1371/journal.pone.0007429 (PMC2759537; doi:10.1371/journal.pone.0007429)
Supplement: Table S1 — Effect of inhibiting FN matrix assembly on PSM cell protrusive and motile activity, elongation and alignment, and probability of egressing. Values presented are averages ±95% confidence intervals. (0.07 MB DOC) [file pone.0007429.s002.doc]

**Supporting Information**

**Table SI**. Effect of inhibiting FN matrix assembly on PSM cell protrusive and motile activity, elongation and alignment, and probability of egressing. Values presented are averages ± 95% confidence intervals.

|  | **Parameter measured** | **Control embryos (BSA)** | | | | **Experimental embryos (70 kDa)** | |  |
| --- | --- | --- | --- | --- | --- | --- | --- | --- |
|  | Pseudopodia (/90min) | 4.5±0.27 | | | | 4.6±0.25 | |  |
|  | Track length (μm/90min) | 39.6±1.71 | | | | 37.6±2.16 | |  |
|  | Displacement (μm/90min) | 4.8±0.61 | | | | 5.5±0.76 | |  |
|  |  | s0*^1^ | | sII*^2^ | | “s0”*^3^ | |  |
|  |  | caudal | rostral | caudal | rostral | caudal | rostral |  |
|  | Cell length (μm) | 28.6±2.5 | 24.8±2.1 | 32.5±3.2 | 28.6±2.4 | 20.7±1.5 | 24.1±2.3 |  |
|  | Centripetal angle (º) | 17.6±3.9 | 29.4±5.7 | 10.2º±2.3 | 11.9.º±2.1 | 31.9º±9.1 | 34.0º±7.9 |  |
|  | Initial cell position: | Periphery | | In core | | Periphery | In core |  |
|  | % epithelialized cells | 93.8% | | 94.6% | | 47.8% | 28.6% |  |
|  | Relative somitocoel volume^*4^  Cell density ratio^*5^ | 0.065±0.010 0.826±0.029 | | | | 0.128±0.026 0.954±0.041 | |  |
|  |  |

*****^1^ s-II +3-4h somite (see Figure 3B)

*****^2^ s-II +6h somite (see Figure 3B)

*****^3^ s-II +6h somite (see Figure 3B); in treated embryos, “s0” somites should have progressed to sII stage.

^*4^ (Volume of somitocoel)/(volume of the whole somite)(see Figure 5C).

^*5^ (DNA labeling in somitocoel)/(DNA labeling in epithelium)(see Figure 5D).
